# Immuno-Hematological Profiles of HIV-Positive Patients Stratified by CD4^+^ T-Cell Counts: Toward Identifying a Surrogate Hematological Marker for Immune Suppression Severity

**DOI:** 10.3390/diagnostics15232976

**Published:** 2025-11-24

**Authors:** Ahmad F. Arbaeen, Mohammad Shahid Iqbal, Radi Alsafi, Hibbah Al Masmoum, Alaa Qadhi, Waheeb Alharbi, Ahmad M. Alharbi, Khalid Kedwa, Mohammad H. Albeshri, Mohammed S. Alameer

**Affiliations:** 1 Department of Clinical Laboratory Sciences, Faculty of Applied Medical Sciences, Umm Al-Qura University, P.O. Box 715, Makkah 21955, Saudi Arabia; 2 Medicine Program, Department of Pathology, Batterjee Medical College, P.O. Box 6231, Jeddah 21442, Saudi Arabia; shahid.iqbal@bmc.edu.sa; 3 Clinical Nutrition Department, Faculty of Applied Medical Sciences, Umm Al-Qura University, P.O. Box 715, Makkah 21955, Saudi Arabia; ahqadhi@uqu.edu.sa; 4 Department of Physiology, Faculty of Medicine, Umm Al-Qura University, P.O. Box 715, Makkah 21955, Saudi Arabia; wdharbi@uqu.edu.sa; 5 Department of Clinical Laboratories Sciences, College of Applied Medical Sciences, Taif University, P.O. Box 11099, Taif 21944, Saudi Arabia; a.alharbii@tu.edu.sa; 6 Medical Biotechnology, Regional Laboratory, Makkah 21955, Saudi Arabia; kkeedw@moh.gov.sa; 7 Department of Hematology, Regional Laboratory, Makkah 21955, Saudi Arabia; malbshri@moh.gov.sa (M.H.A.); malhashmialamer@moh.gov.sa (M.S.A.)

**Keywords:** CD4^+^ T-cell count, immunosuppression, HIV, hematologic parameters, anemia

## Abstract

**Background/Objectives**: The CD4^+^ T-cell count is a primary indicator of immune status in HIV-positive patients. Rapid identification of immune suppression severity may improve clinical decision-making and triage. The relationship between CD4^+^ counts and other immunologic and hematologic markers, however, is not well characterized, especially in resource-limited settings. The objectives of this study were to classify HIV-positive patients by CD4^+^ T-cell count, compare hematologic and immunologic markers across severity groups, assess correlations between CD4^+^ and other variables, and evaluate routine blood tests’ potential to serve as surrogate indicators of immune status. **Methods**: A retrospective cohort of 229 HIV-positive patients from the Regional Laboratory in Makkah, Saudi Arabia, was stratified into three groups: severe (<200 cells/mm^3^), moderate (200–500 cells/mm^3^), and preserved (>500 cells/mm^3^). Hematologic (RBC, Hb, Hct, ESR, WBC, and lymphocytes) and immunologic (CD3, CD4, CD8, B, NK cells, and CD4/CD8 ratio) data were analyzed using ANOVA and Pearson correlation. **Results**: Significant group differences were observed in RBC, Hb, Hct, ESR, and lymphocyte counts (*p* < 0.001). CD4^+^ counts correlated positively with CD3 (r = 0.76), B cells (r = 0.63), and CD8^+^ (r = 0.41) and negatively with ESR (r = –0.37). Over 75% of the patients had disrupted CD4/CD8 ratios, and one-third of the severely immunosuppressed patients showed abnormal B- and NK-cell counts. **Conclusions**: Routine hematologic markers reflect immune suppression severity and can serve as accessible, low-cost tools for monitoring HIV-positive patients in resource-limited settings. Integrating these parameters into immune monitoring may enhance early assessment and provide regional benchmarks for clinical evaluation.

## 1. Introduction

Human immunodeficiency virus (HIV) infection and its advanced stage, acquired immunodeficiency syndrome (AIDS), remain significant global public health challenges. Since the early 1980s, HIV has caused millions of deaths worldwide [1,2]. HIV infection is characterized by progressive depletion of CD4^+^ T-lymphocytes, resulting in impaired cell-mediated immunity and heightened susceptibility to opportunistic infections and certain malignancies. In most cases, AIDS typically develops within 5 to 10 years of HIV infection, contributing to elevated mortality rates [3,4,5,6]. Sub-Saharan Africa bears the highest global burden, accounting for nearly two-thirds of all individuals living with HIV. Other regions, including the Middle East and North Africa (MENA), have reported rising incidence rates and persistent underdiagnosis. Although AIDS-related mortality has declined, the MENA region remains among the few areas where new HIV infections have not significantly decreased over the past decade, as indicated by surveillance and programmatic reviews [7,8]. The first documented case of HIV in Saudi Arabia occurred in 1984 [9].

Although the introduction of Highly Active Antiretroviral Therapy (HAART) has transformed HIV into a chronic but manageable condition, the infection still accounts for significant global morbidity and mortality, especially in regions with limited resources [10,11]. When first introduced in the mid-1990s, antiretroviral therapy (ART) was largely restricted to individuals with advanced HIV infection, owing to its expensiveness, considerable toxicity, and limited drug availability. Early World Health Organization guidelines recommended treatment initiation only for patients with CD4^+^ counts of 200 cells/µL or lower or in the context of stage III/IV AIDS-defining conditions [12].

As evidence demonstrating the benefits of earlier initiation of therapy accumulated, treatment thresholds progressively increased to ≤50 and subsequently to ≤500 cells/µL by the late 2000s [13,14]. The most recent World Health Organization guidelines (2024) now recommend immediately initiating ART for all individuals diagnosed with HIV, irrespective of CD4^+^ count or clinical stage, ideally on the same day of diagnosis whenever feasible [15]. This shift reflects both clinical benefits, including earlier immune recovery, reduced incidence of opportunistic infections, and lower mortality, as well as public health benefits, such as treatment-as-prevention and reduced viral transmission. Highly Active Antiretroviral Therapy (HAART) suppresses viral replication, restores immune function, and lowers transmission risk, thereby forming the cornerstone of global HIV control strategies [8,10,15]. Robust evidence from large, randomized trials, particularly the START and TEMPRANO studies, confirmed that initiating ART at higher CD4^+^ counts significantly reduces both morbidity and mortality, ultimately provoking the WHO to adopt the universal ‘treat all’ strategy [11,16].

Even though ART initiation is no longer contingent upon CD4 counts, CD4 monitoring remains necessary for disease staging, defining advanced HIV (CD4 < 200), guiding prophylaxis with respect to opportunistic infections, and prognostication [10,15]. CD4 and hematological markers continue to offer valuable guidance when viral load testing is unavailable, despite being the gold standard for monitoring treatment response [16,17].

Even with these gains, many nations continue to face hurdles such as late presentation, stigma, treatment resistance, and co-infections (especially tuberculosis, hepatitis B, and hepatitis C) [18]. Access to CD4 and viral load monitoring is also limited by gaps in diagnostic facilities, especially in countries with low and mid-level incomes. These realities have fueled research into using hematological and biochemical indicators such as hemoglobin (Hb), total lymphocyte count (TLC), hematocrit (Hct), and platelets as surrogate markers of immunological state and disease development [12,11,16,19]. By utilizing these surrogate markers, healthcare systems can offer immediate and practical solutions to overcome these barriers. For instance, hematologic testing can be conducted rapidly and at a lower cost, mitigating the delay caused by unavailable CD4 testing facilities. Additionally, accessible surrogate markers can help reduce stigma associated with frequent, visible testing and provide patients with more manageable and discrete methods for monitoring their health.

Moreover, many studies categorize patients dichotomously based on CD4^+^ count (e.g., <200 vs. ≥200 cells/mm^3^), an approach that may overlook important gradations in immune status [20]. There is also a lack of region-specific data from Middle Eastern populations, where genetic, demographic, and healthcare-related factors may shape distinct patterns of immune and hematological dysregulation.

Monitoring CD4^+^ T-cell counts remains a cornerstone in assessing disease progression and treatment response [21]. However, immunologic markers alone may not capture the full spectrum of systemic alterations associated with HIV, particularly in settings with limited access to advanced immunophenotyping. In addition to its impact on immune cells, HIV has substantial effects on hematopoiesis and bone marrow function, often resulting in anemia, leukopenia, thrombocytopenia, and increases in markers of inflammation, such as the erythrocyte sedimentation rate (ESR) [22,23]. These hematologic abnormalities are not only common but may also serve as early indicators of disease severity and prognosis [24]. Nevertheless, most studies assess either immunologic or hematologic parameters in isolation, limiting their clinical utility.

This study examines gender distribution and HIV trends in Saudi Arabia and provides an integrated analysis of immunological and hematological parameters among HIV-positive patients stratified by three CD4^+^ T-cell count categories. The objective is to determine whether routinely available hematologic markers can serve as surrogate indicators of immune suppression severity, thereby supporting patient management in resource-limited settings.

## 2. Materials and Methods

Study design: A retrospective cohort study was conducted using demographic and laboratory data retrieved from the records of the Regional Laboratory in Makkah, Saudi Arabia, for 229 confirmed HIV-positive cases dating from March 2019 to November 2024. Data on ART status and duration of infection were not uniformly available across records and therefore could not be reliably included in the analysis. Diagnosis was based on standard tests, including rapid antibody testing, fourth-generation HIV Ag/Ab Combo ELISA assays, and confirmatory testing via Western Blot or differentiation assays.

CD4^+^ T-cell count was the sole criterion for immunosuppression stratification. Patients (≥18 years) were classified into three groups based on CD4^+^ levels:

- Group I (severe immunosuppression)—CD4^+^ < 200 cells/mm^3^
- Group II (moderate immunosuppression)—CD4^+^ 200–500 cells/mm^3^
- Group III (preserved immunity)—CD4^+^ > 500 cells/mm^3^

Exclusion criteria: Patients with incomplete demographic and laboratory data or with known hematologic malignancies were excluded.

Data were extracted from the regional lab’s electronic medical record and laboratory information systems. The parameters collected included demographics like age, gender, and laboratory data, which included.

- Hematologic data, namely, Red Blood Cell (RBC) counts, Hb, Hct, platelet counts, mean platelet volume (MPV), total WBC counts, the erythrocyte sedimentation rate (ESR), and lymphocyte counts (absolute and relative);
- Immunologic data, namely, CD3^+^, CD4^+^, CD8^+^, B lymphocytes, Natural Killer (NK) cells, and CD4/CD8 ratios.

Co-infections routinely screened in the clinical database included hepatitis B virus (HBV), hepatitis C virus (HCV), syphilis, and tuberculosis Co-infection status was recorded as a binary variable (present/absent).

Flow cytometric analysis was performed using BD FACSCanto II (BD Biosciences, San Jose, CA, USA). Whole blood samples were stained with BD Multitest 6-color CD3/CD4/CD8/CD45/CD16 + CD56/CD19 reagents and analyzed using FACS-Canto software. Absolute counts were derived using the single-platform method with BD Trucount™ tubes. Hematologic tests were conducted using a Sysmex XN-series analyzer, and ESR was measured using the Westergren method.

Reference ranges for lymphocyte subsets were defined according to institutional standards: CD3^+^ T-cells (570–2400 cells/µL), CD4^+^ T-helper cells (400–1800 cells/µL), CD8^+^ cytotoxic T-cells (210–1200 cells/µL), CD19^+^ B-cells (91–610 cells/µL), and NK cells (CD16^+^/CD56^+^, 78–470 cells/µL). An inverted CD4/CD8 ratio was defined as <1.0 (normal range 0.8–3.9). Lymphocyte event counts do not have an established reference range and were therefore interpreted descriptively.

Statistical analysis was performed using IBM SPSS Statistics version 26. Continuous variables were expressed as means ± standard deviations. Group comparisons were made using ANOVA and post hoc Tukey tests. Categorical variables were compared using the chi-square test. Pearson correlation was used to assess relationships between CD4^+^ counts and other laboratory parameters. A *p*-value < 0.05 was considered statistically significant. An inverted CD4/CD8 ratio was defined as <1.0. Group differences were assessed using one-way ANOVA with Tukey’s post hoc test.

The study protocol was approved by the Institutional Review Board (IRB) of the Regional Research Ethics Committee, Makkah Health Affairs (Approval No.: H-02-K-076-1024-1198; approved on 31 October 2024). All patient data were anonymized to ensure confidentiality, and the study was conducted in accordance with the principles outlined in the Declaration of Helsinki.

## 3. Results

Among the 229 individuals living with HIV in this cohort, the mean age was 41.9 ± 11.9 years, and men constituted 79% of the participants (Table 1). Hematological parameters showed that 25.3% had hemoglobin deficiency, and 38% had reduced hematocrit levels. Normal platelet counts were observed in 91.7% of the participants, while 78.5% had elevated ESRs. Regarding white blood cells, 80.5% were within the normal range, and 16.6% were below normal. For lymphocyte parameters, 49.2% had higher-than-normal relative lymphocyte percentages, and 26.3% had increased absolute lymphocyte counts. Immunologically, 44.5% of the patients had low CD4^+^ counts, 22.7% had low CD8^+^ counts, and 27.5% had reduced NK cell levels. The CD4/CD8 ratio was abnormal in 85.1% of the participants.

**Figure 1:**
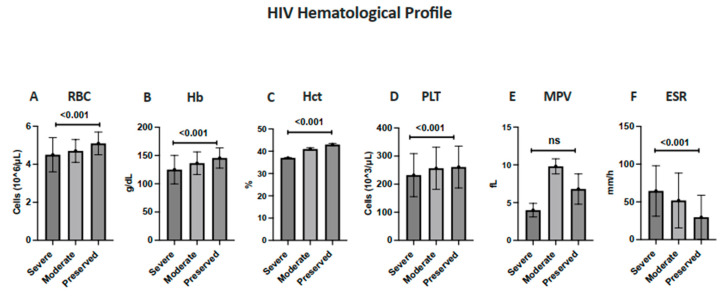
Hematologic and immunologic profiles of HIV-positive patients stratified by disease severity based on CD4^+^ T-cell counts. Mean (±SD) values of hematologic parameters—(**A**) RBC, (**B**) hemoglobin (Hb), (**C**) hematocrit (Hct), (**D**) platelets (PLT), (**E**) Mean platelet volume (MPV) and (**F**) Erythrocyte sedimentation rate (ESR)—are compared among patients with severe, moderate, and preserved immunity. Statistical significance was evaluated using the Kruskal–Wallis test, with *p* < 0.05 considered significant.

The patients were divided into the following three groups according to CD4^+^ counts: severe, moderate, and preserved immunity (as shown in Figure 1). Red cell indices (RBC, Hb, and hematocrit) increased progressively across CD4^+^ strata (all being statistically significant, with *p* < 0.001). Mean RBC and hemoglobin values were lowest in the severely immunosuppressed group and highest in the preserved group; in contrast, ESR demonstrated the opposite pattern, being significantly elevated among patients with severe immunosuppression relative to the other groups (*p* < 0.001). Mean platelet counts did not differ significantly across groups (*p* = 0.432).

**Figure 2:**
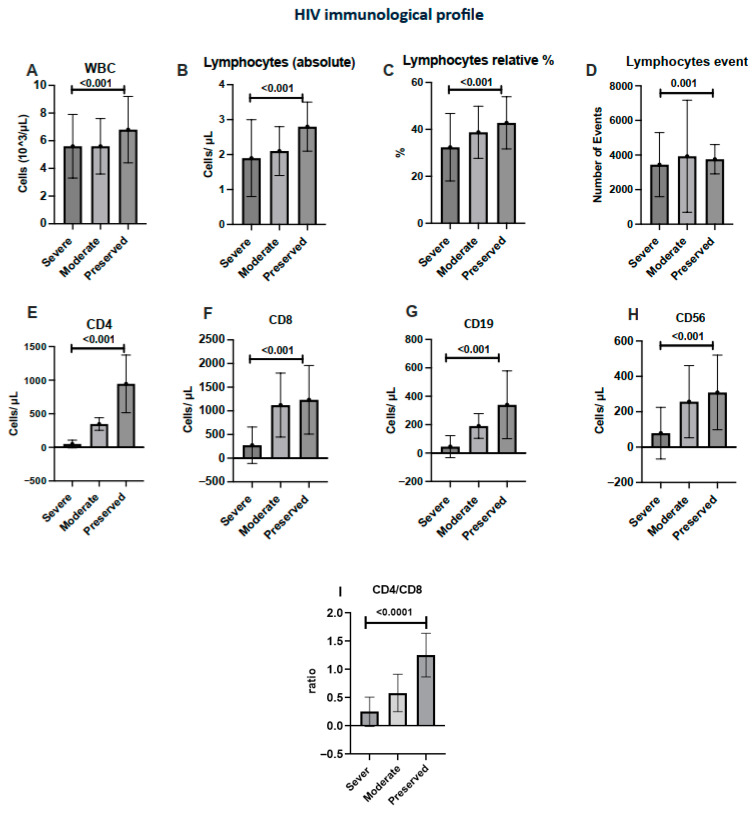
Immunologic profiles of HIV-positive patients stratified by disease severity according to CD4^+^ T-cell count. Mean (±SD) values of hematologic parameters—(**A**) Total WBC count; (**B**) Lymphocytes (absolute); (**C**) Lymphocytes (relative %); (**D**) Lymphocyte event count; (**E**) CD4⁺ T-cell count; (**F**) CD8⁺ T-cell count; (**G**) CD19⁺ B-cell count; (**H**) CD56⁺ NK-cell count; (**I**) CD4/CD8 ratio.Statistical significance was evaluated using the Kruskal–Wallis test with Dunn’s post-hoc comparisons; *p* < 0.05 was considered significant.

In the immunologic profile, as shown in Figure 2, patients with preserved immunity displayed markedly higher counts of CD4, CD8, CD19 (B lymphocytes), and CD56 (NK cells) compared with those with severe immunosuppression (with all values being statistically significant (*p* < 0.001)). Intergroup differences were analyzed using the Kruskal–Wallis test, with *p* < 0.05 considered statistically significant (Table 2).

Among the 229 patients, 73 had severe immunosuppression, 44 had moderate immunosuppression, and 112 had preserved immunity (Table 2). Red cell parameters showed a clear improvement with higher CD4^+^ counts: abnormal RBC values were observed in 38.8% of the severe group, 42.9% of the moderate group, and 13.6% of the preserved group. Hemoglobin deficiency was noted in 45.2%, 29.5%, and 10.7%, respectively, while abnormal hematocrit levels were most frequent in the severe group (57.5%) and least frequent in the preserved group (23.2%). An elevated ESR was found in 96.8% of patients with severe immunosuppression, 76.2% of patients in the moderate group, and 59.3% of patients in the preserved group. White blood cell abnormalities decreased progressively from 31.5% in the severe group to 9.8% in the preserved group. Relative lymphocyte abnormalities were observed in 43.4%, 53.8%, and 67.0% of patients across the groups, while abnormal absolute lymphocyte counts were 41.1%, 14.3%, and 38.2%, respectively.

For immunologic parameters, abnormal CD3 levels occurred in 76.7% of the severe group and 37.5% of the preserved group. All patients with severe immunosuppression (100%) had abnormal CD4^+^ counts; this figure can be compared with 65.9% in the moderate group and 5.4% in the preserved group. Abnormal CD8^+^ values were found in 71.2%, 31.8%, and 48.2% of patients across the respective groups, and reduced NK cell levels were more frequent in the severe group (75.3%) than in the preserved group (22.3%). Abnormal B-lymphocyte levels were noted in 86.3%, 9.1%, and 20.5% of patients in the severe, moderate, and preserved groups, respectively.

The CD4/CD8 ratio differed significantly across immune-suppression categories, increasingly progressively when moving from the severe to moderate and preserved immunity groups (*p* < 0.0001; Figure 2I). An inverted CD4/CD8 ratio was present in 86.3% of the severe group, 97.7% of the moderate group, and 58.9% of the preserved group.

The distribution of co-infections was similar across groups, with no significant enrichment in the severe immunosuppression category (17%, 14%, and 15% in the severe, moderate, and preserved immunity groups, respectively (*p* > 0.05)).

Table 3 summarizes the correlations between CD4^+^ T-cell counts and various hematologic and immunologic parameters, as assessed using Pearson correlation analysis. CD4^+^ count showed significant positive correlations with major hematologic indices, including RBC (r = 0.32, *p* < 0.001), Hb (r = 0.318, *p* < 0.001), Hct (r = 0.338, *p* < 0.001), WBC (r = 0.289, *p* < 0.001), and absolute lymphocyte count (r = 0.505, *p* < 0.001). However, a significant negative correlation was found between CD4^+^ and ESR (r = –0.369, *p* = 0.001). Among immune cell subsets, strong positive correlations with CD3^+^ (r = 0.762, *p* < 0.001), B-lymphocytes (r = 0.63, *p* < 0.001), CD8^+^ (r = 0.411, *p* < 0.001), NK cells (r = 0.358, *p* < 0.001), and the CD4/CD8 ratio (r = 0.555, *p* < 0.001) were observed.

## 4. Discussion

Despite significant advances in ART, HIV remains a major global health challenge, contributing to considerable morbidity and mortality. In Saudi Arabia, the overall prevalence is relatively modest; however, underdiagnosis, stigma, and co-infections continue to impede effective treatment [25]. Although current WHO guidelines recommend prompt ART initiation for all individuals, CD4^+^ counts and alternative hematologic indicators remain essential for disease staging and monitoring. In many low- and middle-income countries, including several regions within the MENA, access to CD4^+^ and viral load testing remains limited. To address these gaps, the WHO has endorsed the use of hematological surrogates, Hb concentration, and body mass index (BMI) to guide treatment initiation when CD4^+^ testing is unavailable [12,17,19]. Integrating these surrogate markers into national guidelines could significantly streamline the decision-making process and improve care in resource-limited settings.

The demographic profile of this cohort, characterized by a mean age of ~42 years and male predominance, aligns with national and regional reports. Early surveillance (1984–2003) showed most cases occurred among men of working age, primarily in urban centers such as Jeddah, Riyadh, and Dammam [9]. Later data confirmed persistent male predominance, with infections concentrated in young adults, although the proportion of women has gradually increased [26]. Hospital-based cohorts in Riyadh similarly reported median ages in the late 30 s to early 40 s, findings consistent with our findings [3].

We categorized 229 HIV-positive individuals into three groups based on CD4^+^ T-cell counts, namely, severe (<200 cells/mm^3^), moderate (200–500 cells/mm^3^), and preserved (>500 cells/mm^3^), and evaluated their immune-hematologic profiles. The hematologic and immunologic parameters of each group were compared to assess variations across disease stages. Anemia was one of the most prevalent abnormalities in this cohort, affecting 25.3% of all patients and more than half of those with severe immunosuppression. Hb, Hct, and RBC counts all demonstrated significant positive correlations with CD4^+^ levels, indicating that hematologic deterioration parallels immune suppression. Overall, RBC counts declined by 28.4%, with the steepest reductions observed in patients with severe disease. Similar relationships have been observed in studies from Nigeria, Ethiopia, and Pakistan, where patients with CD4^+^ counts <200 cells/µL had significantly lower Hb levels, particularly in the presence of tuberculosis or HCV co-infection [4,5,13,11,19,27].

Moderate-to-severe anemia has been reported to be an independent predictor of mortality among ART-naïve patients in Indonesia [10]. The pathophysiological mechanisms involve direct HIV-mediated marrow suppression, cytokine-induced inhibition of erythropoiesis, nutritional deficiencies, and opportunistic infections [4,5,6,27]. Collectively, these findings support the role of Hb as a robust and cost-effective surrogate marker for disease severity, particularly where access to CD4^+^ testing is constrained.

In this study, it was found that platelet counts did not differ significantly among CD4^+^ groups, indicating preserved thrombopoiesis. In contrast to international findings showing high thrombocytopenia rates in advanced HIV cases [14,18,28], most patients in this study maintained normal platelet levels (91.7%), with only 4.4% exhibiting thrombocytopenia, possibly reflecting regional co-infection patterns, supportive care practices, or earlier ART initiation. These findings highlight the importance of regional context in interpreting hematologic manifestations of HIV and suggest that improved clinical management may mitigate platelet suppression commonly observed in the advanced stages of this disease.

Our findings of strong positive correlations between Hb and CD4^+^ are consistent with those of previous studies [4,5,10,13,11,19,27,28,29]. Moreover, anemia and thrombocytopenia have been found to be common in severe immunosuppression and to serve as independent predictors of mortality, as reported in a Libyan cohort [28]. Moderate correlations were also found between CD4^+^ and Hb, RBC, and WBC, supporting the relationship between immune status and hematologic function. Nigerian cohorts have further suggested that Hct can serve as a surrogate for CD4^+^ <200, based on strong associations with Hb and Hct [19,29]. Such findings highlight that CD4^+^ decline reflects a broader systemic immunehematological disturbance. The gradual normalization of Hb, ESR, WBC, and lymphocyte counts across CD4^+^ strata in this study reinforces their potential as cost-effective surrogates, particularly in resource-limited settings. There was a negative correlation between CD4^+^ counts and the ESR (r = −0.369, *p* = 0.001). An elevated ESR was present in 78.5% of patients with severe immunosuppression. While nonspecific, an increased ESR in advanced HIV reflects systemic inflammation and opportunistic infections. Similar associations have been documented in Ethiopian and Nigerian cohorts [5,19]. Our findings support using the ESR as an inexpensive, non-specific inflammatory marker that mirrors immune activation and disease progression.

In this study, 16.6% of the patients had leukopenia, with a higher prevalence among those with severe immunosuppression. Absolute lymphocyte counts (ALCs) showed a strong positive correlation with CD4^+^ levels (r = 0.505, *p* < 0.001), consistent with studies from Uganda and Ethiopia reporting moderate correlations between CD4^+^ and TLC (r = 0.33–0.71) [12,11]. Absolute lymphocyte counts and percentages were significantly lower in the patients with severe immunosuppression, and nearly half of the cohort had reduced relative lymphocyte percentages. Thus, lymphocyte-based measures represent cost-effective supplements to CD4^+^ testing, particularly where diagnostic resources are limited [12,17]. Although the WHO initially recommended that a TLC ≤ 1200/µL can serve as a surrogate for CD4^+^ < 200, this marker’s limited sensitivity and specificity have been well-documented [11,17,19]. While TLC alone lacks sufficient diagnostic precision, combining it with Hb and lymphocyte percentage significantly improves predictive accuracy [12,13,11,16,17,19]. A notable Saudi contribution is the three-parameter score developed at King Abdulaziz University Hospital (Jeddah), incorporating Hb, TLC, and lymphocyte percentage. This model effectively predicted CD4^+^ <200 cells/µL and provided a cost-efficient indicator of immune status in resource-limited settings [16]. Similar findings from Nigeria, Ethiopia, and Pakistan highlight moderate associations between CD4^+^ counts and TLC or Hb, underscoring their role as supplements rather than substitutes [13,11,19]. Extensive research has evaluated the ability of the TLC to serve as a surrogate for CD4^+^ testing. TLC thresholds between 1200 and 2300/µL have been proposed to predict CD4^+^ counts <200 or <350 cells/µL [11,19]. Although the TLC alone lacks sufficient accuracy, its utility improves when combined with other parameters [12].

Patients with severe immunosuppression had the lowest CD4^+^ counts and CD4/CD8 ratios, while those with preserved immunity exhibited progressive restoration of these indices. CD8^+^ expansion and CD4/CD8 inversion (<1) were present in 85% of the cohort, consistent with classical HIV-associated immune dysregulation [4,6]. The significant positive associations between CD4^+^ counts and CD3^+^ (r = 0.76), B lymphocytes (r = 0.63), and NK cells highlight the coordinated impairment and recovery of multiple immune lineages. These results mirror both regional and international findings and reinforce the notion that hematologic recovery parallels immunologic restoration following ART [4,10,12,16].

Our findings underscore the clinical utility of routine hematological and biochemical markers in HIV monitoring. Even in the “treat all” ART era, CD4^+^ counts remain essential for staging and prophylaxis against opportunistic infections [15]. Where CD4^+^ or viral load testing is unavailable, simple measures such as hemoglobin (Hb), hematocrit (Hct), and lymphocyte counts can serve as inexpensive and accessible surrogates [16,19]. These indicators could strengthen HIV care in resource-limited areas if incorporated into national guidelines. By providing comprehensive immune–hematologic baseline data from a Middle Eastern HIV cohort, this study addresses an important regional knowledge gap and highlights the need for individualized care models within Saudi national HIV programs.

The Saudi National AIDS Program is encouraged to consider piloting a hematology-based screening protocol to harness these findings. Such an initiative could not only improve early detection and monitoring in resource-limited environments but also serve as a model for neighboring regions. The timely adoption and implementation of this approach could accelerate public health responses to HIV, transforming evidence into actionable policy for the betterment of patient care across Saudi Arabia.

Hematological indicators serve as practical alternatives to CD4^+^ and viral load monitoring in settings where these tools are limited. Incorporating these models into national protocols could improve early detection and monitoring at peripheral clinics. Despite the disease’s general population prevalence of <0.1%, up to 90% of HIV cases in Saudi Arabia may remain undiagnosed due to stigma, limited awareness, and insufficient targeted screening [25,30]. By 2018, the Ministry of Health estimated an incidence of 3 per 10,000 and a cumulative total of more than 23,000 cases [8]. This study presents one of the first detailed immune–hematologic characterizations of HIV-positive patients in Saudi Arabia, filling a regional knowledge gap. Strong associations were identified between CD4^+^ counts and basic hematological markers, including hemoglobin, hematocrit, lymphocyte counts, and ESR. These parameters serve as inexpensive and accessible indicators of immune suppression, complementing CD4^+^ and viral load testing, particularly in resource-limited settings. Incorporating these markers into national HIV care protocols could enhance monitoring and support the implementation of timely interventions.

The sample size and stratification by CD4^+^ counts enabled meaningful comparisons, and the combined analysis of hematologic and immunologic parameters added clinical relevance. However, this study’s cross-sectional design limits causal interpretation, and recruitment from a single region may have affected generalizability. Potential confounders such as nutritional status and co-infections were not fully controlled, although the comparable distribution of co-infections across CD4^+^ categories suggests that co-infection burden was unlikely to confound the main hematologic or immunologic patterns. Additionally, incomplete documentation of ART status and duration of infection further constrained adjustment for key clinical modifiers. In people with HIV, anemia may also arise from nutritional deficiencies, chronic inflammation, ART-related marrow suppression, or opportunistic infections, which could influence hemoglobin, hematocrit, and RBC levels independently of CD4-defined immunosuppression. Despite these limitations, the findings align with international data and underscore the utility of routine hematological markers as adjuncts to CD4^+^ monitoring in resource-limited settings.

## 5. Conclusions

Based on our findings and the supporting literature, the most dependable surrogate indicators of CD4^+^ status are (1) Hb, Hct, and RBC, which strongly correlate with disease severity; (2) absolute TLC, a proven substitute where CD4^+^ testing is unavailable; (3) relative lymphocyte percentage, which enhances predictive value when combined with Hb and TLC; and (4) ESR, which demonstrates a negative correlation with CD4^+^ and serves as a marker of advanced disease and systemic inflammation. These findings offer practical criteria for clinical evaluation in resource-limited settings and demonstrate that routine hematologic measures can reflect the underlying immunological status.

## Figures and Tables

**Table 1:** Baseline characteristics of the participants (*n* = 229).

Parameter	Mean ± SD and Frequency (Percentage)
Age (Years)	41.9 ± 11.9
Gender	
Male	79%
Female	21%
RBC	4.8 ± 0.8 × 10^6^/µL
Normal	71.6%
Deficiency	28.4%
Hemoglobin (Hb)	137.5 ± 22.6 g/dL
Normal	74.7%
Deficiency	25.3%
Hematocrit (Hct)	0.4 ± 0.08 L/L
Normal	62%
Deficiency	38%
ESR	49.5 ± 35.7 mm/h
Normal	21.5%
Higher than normal	78.5%
Platelets	250.9 ± 76.1
Lower than normal	4.4%
Normal	91.7%
Higher than normal	3.9%
MVP	9.9 ± 0.9
Normal	74.7%
Higher than normal	25.3%
WBC	6.2 ± 2.4 × 10^3^/µL
Lower than normal	16.6%
Normal	80.5%
Higher than normal	2.6%
Relative Lymphocytes %	39 ± 12.9
Lower than normal	8.2%
Normal	42.6%
Higher than normal	49.2%
Lymphocytes (Absolute)	2.4 ± 1 × 10^3^/µL
Lower than normal	8.3%
Normal	65.4%
Higher than normal	26.3%
Lymphocyte Event	3690.6 ± 1855.7 cells/µL
CD3	1502.4 ± 1105.1 × 10^3^/µL
Lower than normal	24.9%
Normal	55.5%
Higher than normal	19.7%
CD4	544.9 ± 506.7 cells/µL
Lower than normal	44.5%
Normal	52.8%
Higher than normal	2.6%
CD8	906 ± 760.3 cells/µL
Lower than normal	22.7%
Normal	47.6%
Higher than normal	29.7%
NK	225.8 ± 216.4 cells/µL
Lower than normal	27.5%
Normal	59.8%
Higher than normal	12.7%
B Lymphocyte	217.4 ± 219.3 cells/µL
Lower than normal	32.3%
Normal	60.7%
Higher than normal	7%
4/8 Ratio	0.6 ± 0.5
Normal	24.9%
Abnormal	85.1%

Data are presented as means ± standard deviations (SDs) for continuous variables and as numbers (percentages) for categorical variables.

**Table 2:** Frequency of the patients’ characteristics based on CD4^+^ disease severity.

Parameter	Severe Immunosuppression		Moderate Immunosuppression		Preserved Immunity	
	Normal	Abnormal	Normal	Abnormal	Normal	Abnormal
RBC	44 (60.3%)	29 (39.7%)	25 (57.1%)	19 (42.9%)	96 (86.4%)	16 (13.6%)
Hb	40 (54.8%)	33 (45.2%)	31 (70.5%)	13 (29.5%)	100 (89.3%)	12 (10.7%)
Hct	31 (42.5%)	42 (57.5%)	25 (56.8%)	19 (43.2%)	86 (76.8%)	26 (23.2%)
ESR	2 (3.2%)	71 (96.8%)	10 (22.8%)	34 (76.2%)	46 (40.7%)	66 (59.3%)
Platelets	65 (89.0%)	8 (11.0%)	41 (93.2%)	3 (6.8%)	104 (92.9%)	8 (7.1%)
MPV	55 (75.3%)	18 (24.7%)	33 (75.0%)	11 (25.0%)	83 (74.1%)	29 (25.9%)
WBC	50 (68.5%)	23 (31.5%)	34 (77.3%)	10 (22.7%)	101 (90.2%)	11 (9.8%)
Lymphocytes (Relative %)	41 (56.6%)	32 (43.4%)	20 (46.2%)	24 (53.8%)	37 (33.0%)	75 (67.0%)
Lymphocytes (absolute)	43 (58.9%)	30 (41.1%)	37 (85.7%)	7 (14.3%)	69 (61.8%)	43 (38.2%)
CD3	17 (23.3%)	56 (76.7%)	40 (90.9%)	4 (9.1%)	70 (62.5%)	42 (37.5%)
CD4	0 (0.0%)	73 (100%)	15 (34.1%)	29 (65.9%)	106 (94.6%)	6 (5.4%)
CD8	21 (28.8%)	52 (71.2%)	30 (68.2%)	14 (31.8%)	58 (51.8%)	54 (48.2%)
NK	18 (24.7%)	55 (75.3%)	32 (72.7%)	12 (27.3%)	87 (77.7%)	25 (22.3%)
B-lymphocytes	10 (13.7%)	63 (86.3%)	40 (90.9%)	4 (9.1%)	89 (79.5%)	23 (20.5%)
CD4/CD8 ratio	10 (13.7%)	63 (86.3%)	1 (2.3%)	43 (97.7%)	46 (41.1%)	66 (58.9%)
Co-infection	Yes 19 (17%)/No 93 (83%)		Yes 6 (14%)/No 38 (86%)		Yes 11 (15%)/No 62 (85%)	

Percentages represent the proportion of patients within each CD4^+^ severity category.

**Table 3:** Correlations between CD4^+^ and other factors.

Parameter	r (*p*-Value)
Age	0.01 (0.88)
RBC	0.32 (<0.001)
Hb	0.318 (<0.001)
Hct	0.338 (<0.001)
ESR	−0.369 (0.001)
Platelet	0.198 (0.003)
MPV	−0.099 (0.136)
WBC	0.289 (<0.001)
Lymphocyte relative %	0.295 (<0.001)
Lymphocyte relative	0.276 (<0.001)
Lymphocyte absolute	0.505 (<0.001)
Lymphocyte event	0.058 (0.385)
CD3^+^	0.762 (<0.001)
CD8^+^	0.411 (<0.001)
NK	0.358 (<0.001)
B-lymphocytes	0.63 (<0.001)
4/8 ratio	0.555 (<0.001)

Values are Pearson’s correlation coefficients (r) with corresponding *p*-values. Correlations were assessed using Pearson’s correlation coefficient (r). *p*-values < 0.05 were considered statistically significant.

## Data Availability

The data supporting the findings of this study are available from the Regional Laboratory in Makkah, Saudi Arabia, but restrictions apply to the availability of these data due to privacy and ethical regulations. The data are therefore not publicly available. De-identified data may be made available by the corresponding author upon reasonable request and with permission from the Regional Research Ethics Committee, Makkah Health Affairs.

## Abbreviations

The following abbreviations are used in this manuscript:

AIDS	Acquired Immunodeficiency Syndrome
ALC	Absolute Lymphocyte Count
ANOVA	Analysis of Variance
ART	Antiretroviral Therapy
BMI	Body Mass Index
CD	Cluster of Differentiation
ESR	Erythrocyte Sedimentation Rate
HAART	Highly Active Antiretroviral Therapy
Hb	Hemoglobin
Hct	Hematocrit
HIV	Human Immunodeficiency Virus
IRB	Institutional Review Board
MENA	Middle East and North Africa
NK	Natural Killer (cells)
PLHIV	People Living with HIV
RBC	Red Blood Cell
TLC	Total Lymphocyte Count
WBC	White Blood Cell
WHO	World Health Organization
